# 

**Published:** 1892-11-19

**Authors:** 


					~  ; f November 19thg ]
PRIGF TWHPFNfiF - WW ? WITH EXTRA NURSING SUPPLEMENT,
v, 891 l# i v ii ii inif ionn I if Per Annum, 8s. 6d., post frw.
Vol. XIII. NOV. 19th, 1892. M Monthly Farts. 6d.; post free, 8d.
,or ^ nitt? Aaa??? ^
No. 321. Vol. XIII.] Saturday,
November 19th, 1892.
[Twopence.

				

